# Reconciling Contemporary Approaches to School Attendance and School Absenteeism: Toward Promotion and Nimble Response, Global Policy Review and Implementation, and Future Adaptability (Part 1)

**DOI:** 10.3389/fpsyg.2019.02222

**Published:** 2019-10-15

**Authors:** Christopher A. Kearney, Carolina Gonzálvez, Patricia A. Graczyk, Mirae J. Fornander

**Affiliations:** ^1^Department of Psychology, University of Nevada, Las Vegas, Las Vegas, NV, United States; ^2^Department of Developmental Psychology and Teaching, University of Alicante, San Vicente del Raspeig, Spain; ^3^Department of Psychiatry, University of Illinois at Chicago, Chicago, IL, United States

**Keywords:** school attendance, school absenteeism, truancy, school refusal, school withdrawal, school exclusion, multi-tiered system of supports, response to intervention

## Abstract

School attendance is an important foundational competency for children and adolescents, and school absenteeism has been linked to myriad short- and long-term negative consequences, even into adulthood. Many efforts have been made to conceptualize and address this population across various categories and dimensions of functioning and across multiple disciplines, resulting in both a rich literature base and a splintered view regarding this population. This article (Part 1 of 2) reviews and critiques key categorical and dimensional approaches to conceptualizing school attendance and school absenteeism, with an eye toward reconciling these approaches (Part 2 of 2) to develop a roadmap for preventative and intervention strategies, early warning systems and nimble response, global policy review, dissemination and implementation, and adaptations to future changes in education and technology. This article sets the stage for a discussion of a multidimensional, multi-tiered system of supports pyramid model as a heuristic framework for conceptualizing the manifold aspects of school attendance and school absenteeism.

## Introduction

School attendance and successful graduation from high school or its equivalent have long been recognized as crucial foundational competencies for children and adolescents. Strong school attendance and successful graduation are closely linked to broad, positive outcome variables such as enhanced lifetime earning potential and economic empowerment (Balfanz et al., 2014; Balfanz, 2016), opportunities for higher education and other avenues of adult and career readiness (Darling-Hammond et al., 2014), improved health and reduced death rates (Freudenberg and Ruglis, 2007; Allison and Attisha, 2019), better civic engagement and outcomes (Zaff et al., 2017; DePaoli et al., 2018), and critical thinking, risk aversion, and life skills that impact positive economic and health-based choices (Brunello and De Paola, 2014). In related fashion, strong school attendance and successful graduation may enhance quality of life and buffer against negative mental and physical health outcomes (Rumberger, 2011; U.S. Census Bureau, 2012; Lee et al., 2016).

Conversely, school attendance problems, including school absenteeism, have long been recognized as a critical developmental challenge and limiting factor for children and adolescents (Kearney, 2016). School attendance problems in various forms have been linked to a wide array of academic deficiencies such as reduced educational performance, lower reading and mathematics test scores, fewer literacy skills, grade retention, and school dropout (Bridgeland et al., 2006; Burton et al., 2014; Smerillo et al., 2018). School attendance problems are closely linked as well to internalizing behavior problems such as anxiety, depression (including issues of suicidal behavior and bereavement), and social isolation (Ek and Eriksson, 2013; Pompili et al., 2013; Miller et al., 2015; Finning et al., 2019; Knollmann et al., 2019) as well as externalizing behavior problems such as elevated alcohol, tobacco, marijuana, and other drug use (Henry and Huizinga, 2007; Holtes et al., 2015), risky sexual behaviors (Allison and Attisha, 2019), oppositional defiant and conduct problems (Wood et al., 2012), impaired social functioning and poor relationships with peers (Havik et al., 2015; Gonzalvez et al., 2019), and involvement with the juvenile justice system (Anderson et al., 2016). School attendance problems are connected to myriad adverse childhood experiences such as trauma, school violence and victimization, and medical problems as well (Hutzell and Payne, 2012; Ramirez et al., 2012; Emerson et al., 2016; Hsu et al., 2016; McLean et al., 2017; Stempel et al., 2017; Berendes et al., 2019).

School attendance problems have long-lasting effects even into adulthood, including enhanced risk for marital and psychiatric problems (Hibbett and Fogelman, 1990), non-violent crime and substance use (Henry et al., 2012; Rocque et al., 2017), and occupational problems and economic deprivation (Christenson and Thurlow, 2004; Bridgeland et al., 2006). Students who drop out of high school are 24 times more likely than graduates to experience four or more negative life outcomes (Lansford et al., 2016). The societal outlays for school dropout are substantial as well, including elevated economic costs due to increased crime, incarceration, public assistance, unemployment, and medical coverage as well as reduced mobility, tax revenues, earnings, entrepreneurship, and productivity (Marchbanks et al., 2014; Latif et al., 2015; Levin, 2017).

School attendance problems have no consensus definition (see later section) but lack of school attendance as well as permanent school dropout have been identified as widespread global phenomena with substantial prevalence rates, especially among developing areas such as sub-Saharan and northern Africa and southern and western Asia. Nearly one of five children and adolescents worldwide (17.8%) are out of school, a rate more than doubled among upper secondary school-age youth (36.3%) and elevated among girls and those in low-income countries. Even in Europe and North America, the out-of-school rate is 4.3% (UNESCO Institute for Statistics, 2016). In the United States, the high school graduation rate is 84.1%, the status dropout rate is 6.1%, and the chronic absenteeism rate (federally defined as missing 15+ (8.3%) days of school in one academic year) is 16.0%, a rate elevated among diverse youth, students with disabilities, and high school students (21.1%) (DePaoli et al., 2018; National Center for Education Statistics, 2018; U.S. Department of Education, 2019). As such, school attendance is often viewed as a key linchpin for prevention science and for curbing mental health and other problems in children and adolescents worldwide (Kieling et al., 2011; Catalano et al., 2012).

The substantial impact and prevalence of school attendance and school absenteeism (SA/A) have led researchers across many disciplines to study these phenomena, including those in psychology, education, criminal and juvenile justice, social work, medicine, psychiatry, nursing, epidemiology, public and educational policy, program evaluation, leadership, child development, and sociology, among other professions (Elliot, 1999; Kearney, 2003; Birioukov, 2016). Research in this area has been conducted for over a century, making SA/A among the longest-investigated issues among children and adolescents (Kearney, 2001). This lengthy period of study has led to a plethora of terms and approaches to describe this population, which has led simultaneously to a rich literature base but also to considerable splintering across disciplines and thus a lack of consensus with respect to defining, conceptualizing, classifying, assessing, and addressing SA/A (Kearney, 2016, 2019). Such splintering has likely led to dissemination and implementation barriers regarding empirically based strategies for SA/A (Arora et al., 2016).

## Evolution of Concepts in School Attendance and School Absenteeism

The purpose of this article is to draw upon this rich and disparate literature base to begin to reconcile various contemporary approaches to SA/A and to develop a heuristic framework for conceptualizing this population moving forward. Such a framework is necessary given several needs: to promote school attendance as much as to reduce absenteeism, to respond nimbly to emerging school attendance problems, to inform policy review, to provide general applicability to various jurisdictions and cultures, and to adapt to future and rapid changes in education and technology. As such, a contemporary framework for SA/A will need to be inclusive, flexible, applicable, educational, and pliable.

Efforts to conceptualize SA/A are manifold, in part because of the heterogeneous nature of the constructs and because risk factors for these problems are multilayered and myriad (van der Woude et al., 2017). However, these conceptualization efforts can be grouped generally into categorical and dimensional approaches. Historical efforts to conceptualize SA/A began with categorical terms, dichotomies, and distinctions to try to sort youth with school attendance problems into defined groups in an effort to better understand the mechanisms underlying such behaviors (Kearney, 2001). Categorical approaches broadly aim for within-category homogeneity and between-category qualitative differences (De Boeck et al., 2005), goals that have been somewhat elusive for SA/A (DiBartolo and Braun, 2017).

Other efforts to conceptualize SA/A have focused more on dimensional approaches to better reflect the heterogeneity, fluidity, scalability, and complexity of these constructs (Kearney and Silverman, 1996). Such approaches, described in more detail in later sections, focus on fluid or latent constructs such as attendance profiles, absenteeism severity, risk factors, functions, and interventions that can be arranged along various spectra or continua (Maynard et al., 2012). Dimensional approaches generally aim for within-category heterogeneity and between-category quantitative differences (De Boeck et al., 2005), goals that can also be challenging for SA/A (Heyne et al., 2019).

The juxtaposition of categorical and dimensional approaches to mental health and related challenges has led historically to strong debates about which approach best characterizes a given phenomenon or set of phenomena such as mental disorders (Widiger and Samuel, 2005). Such debate is intensified by the fact that specific taxa for personality and psychopathology are difficult to distinguish even though clinicians and educational and mental health agencies often rely on categorical approaches (Haslam et al., 2012). In addition, mental disorders and psychopathological constructs can be categorically different from normal function in some cases (e.g., psychotic or eating disorder) but not in other cases (e.g., personality disorder, worry), further muddying the classification waters (Ruscio and Ruscio, 2008).

Coghill and Sonuga-Barke (2012) described several avenues for reconciling this debate with respect to mental health and other challenges in children and adolescents. These avenues include replacing categorical with dimensional approaches at various levels or utilizing a mixed approach whereby categories and dimensions are considered alongside one another. With respect to the latter avenue, this could include allowing some phenomena to be described categorically (e.g., autism, endogenous depression) and other phenomena to be described dimensionally (e.g., psychopathy, exogenous depression). Or, in a mixed approach, both categorical and dimensional approaches could be used together within the same class of disorder (e.g., the category of attention-deficit/hyperactivity disorder with dimensions of inattentiveness and hyperactivity/impulsivity). Coghill and Sonuga-Barke (2012) maintained that systems based on both categorical and dimensional approaches can coexist within a single problem by serving different but equally useful purposes.

The next sections of this article (Part 1 of the review) contain brief descriptions of common categorical terms and distinctions as well as dimensional approaches to the study of SA/A. These sections also briefly describe the advantages and disadvantages of each method. In Part 2 of this review, we adopt Coghill and Sonuga-Barke’s (2012) premise that both categorical and dimensional approaches can be applied to a given heterogeneous construct such as SA/A and, indeed, that these approaches are wholly compatible with one another with respect to SA/A. In addition, such compatibilities may be helpful for developing a roadmap for researchers, clinicians, and educators to follow as they work to develop preventiative and nimble responses to SA/A, disseminate research work, and adapt to future changes in education and technology.

## Terminology

As mentioned, school attendance problems have no consensus definition, in part because of the various terms used to describe this population from different disciplines. This section provides general descriptions of common categorical terms utilized in the field, with the strong caveat that considerable controversy and heterogeneity remain even with respect to these characterizations (Kiani et al., 2018). Most broadly, *school attendance* has traditionally referred to a student’s complete in-class physical presence during an academic day and *school absenteeism* has traditionally referred to a student’s complete in-class physical absence during an academic day (Kearney, 2019). School absenteeism is sometimes categorized as *excused* or *unexcused* (or *authorized* or *unauthorized*) in nature, referring to absence due to some legitimate reason such as illness or absence due to some illegitimate reason such as peer association outside of school (Gottfried, 2009). *School attendance problems*, which can include school absenteeism, refer generally to either a collection of different kinds of absences (e.g., late to school/tardiness; skipped class or missed time of day) or to general difficulties attending or getting to school that can involve a wide array of individual and contextual factors (Kearney, 2016). School attendance problems can lead eventually to *school stopout*, which refers to temporary departure from school prior to graduation, and/or s*chool dropout/stayout*, which refers to permanent, premature departure from school prior to graduation (Boylan and Renzulli, 2017).

Several terms in the literature refer generally, though not always, to youth-based school attendance problems, or absences initiated primarily by a child or adolescent, with the caveat that many different risk factor levels (e.g., parent, peer, school) apply to this population. *Truancy* is one of the oldest terms for school attendance problems and refers generally to illegal, unexcused (see later section) school absenteeism. Truancy is a term often utilized by school districts and/or larger entities to construct policies and definitions, such as 10 unexcused absences in a given semester or 15-week period, that trigger some legal, punitive, or administrative consequence (Sutphen et al., 2010). From a research perspective, truancy is often associated as well with delinquency, externalizing behavior problems, and social conditions such as poverty (Zhang et al., 2010).

*School refusal* refers broadly to school attendance problems due to emotional difficulties such as general and social and separation anxiety, worry, distress, and sadness (Elliott and Place, 2019). A related but archaic term, *school phobia*, refers more specifically to fear-based school attendance problems such as avoidance of a specific object at school or related to school (e.g., alarm, animal, bus) that leads to absenteeism (Inglés et al., 2015). *School refusal behavior* refers to a child-motivated refusal to attend school or difficulties remaining in classes for an entire day (Kearney and Silverman, 1990, 1996). School refusal behavior may or may not be related to emotional distress about school, and thus serves as an umbrella term for constructs such as truancy and school refusal.

Other terms in the literature refer to school attendance problems initiated primarily by entities other than the child, again with the caveat that multiple risk factor levels apply to each. *School withdrawal* refers generally to parent-initiated school absenteeism (Kahn and Nursten, 1962; Kearney and Fornander, 2018). Parents or other caregivers may deliberately keep a child home from school for employment or child care purposes, to conceal maltreatment, to protect a child from perceived harm (e.g., school violence or victimization, kidnapping by an ex-spouse), to punish a child, or to mitigate a parent’s separation anxiety or psychopathology due to anxiety, depression, substance use, or other problem, among other reasons (Kearney, 2001).

In addition, *school exclusion* refers generally to school-initiated absenteeism. Such exclusion may involve lawful exclusionary disciplinary practices such as suspension or expulsion for behavior problems or for, ironically, school absenteeism (Maag, 2012). School exclusion practices are often associated with zero tolerance policies regarding certain student behaviors, particularly those related to violence and other dangerous behavior (Theriot et al., 2010). School exclusion may also involve unlawful, unclear, or more nefarious reasons such as sending students (in particular special needs students) home or restricting their ability to attend school without official documentation (McCluskey et al., 2016).

## Categorical Distinctions

Related to these historical terms have been various broad-band and etiologically based categorical dichotomies and distinctions for SA/A. These dichotomies and distinctions have been generally designed to carve out groups of youth with different school attendance problems to help identify causal factors as well as basic treatment direction and scope (Reid, 2013).

### School Refusal-Truancy

An enduring categorical dichotomy has involved school refusal-truancy, which has been historically based on an internalizing-externalizing behavior problem distinction (Young et al., 1990). School refusal is often linked to internalizing difficulties such as anxiety and depression, whereas truancy is often linked to externalizing difficulties such as oppositional and conduct problems (Dembo et al., 2016). In addition, school refusal is sometimes associated with parental knowledge of a child’s absenteeism, whereas truancy is often tied to lack of parental knowledge (Bobakova et al., 2015). School refusal may be more associated with primary or early secondary grades, whereas truancy may be more associated with later secondary grades (Melvin et al., 2017; Pengpid and Peltzer, 2017). School refusal may be more associated with certain family dynamics such as enmeshment, whereas truancy may be more associated with certain family dynamics such as conflict (McConnell and Kubina Jr, 2014; Richardson, 2016).

A main advantage of a school refusal-truancy distinction is its face validity, as some children are clearly anxious and thus avoidant of school whereas some adolescents refuse or decline to attend school without emotional difficulty and with perhaps more delinquency (Berg, 1997; Evans, 2000). The dichotomy carries a significant number of disadvantages, however. First, numerous studies and reviews have demonstrated considerable heterogeneity *within* each construct (Inglés et al., 2015). School refusal is linked to a wide variety of anxiety- and mood-based conditions in addition to fairly broad terms such as emotional distress, avoidance, malingering, dread, worry, fear, somatic complaints, and negative affectivity (e.g., Sibeoni et al., 2018). In addition, truancy is a highly heterogeneous construct with multiple dimensions related to academic status, disability profile, location, race/ethnicity, activities in and out of school, individual-group-orientation, premediated-spontaneous, parental academic involvement, and type and number of classes skipped, among many other variables (Reid, 1999; Chen et al., 2016; Dahl, 2016; Sälzer and Heine, 2016; Keppens and Spruyt, 2017; Maynard et al., 2017). Truancy as a legal construct is also highly variably defined across many jurisdictions (Gentle-Genitty et al., 2015).

Second, many researchers have demonstrated substantial heterogeneity *across* the two constructs. Both school refusal and truancy have been associated, for example, with learning and health difficulties, effects from bullying, social interaction problems, maltreatment, chronic illness, and, of course, missing school (Katz et al., 2016; Lum et al., 2017). In addition, both constructs can be similarly influenced by broader classes of contextual factors related to peers, schools, and communities (Baier, 2016; Sugrue et al., 2016; Burdick-Will et al., 2019). Many historical and statistical studies have also demonstrated either considerable overlap of school refusal and truancy and/or other, large unclassified categories (Torma and Halsti, 1975; Berg et al., 1985; Cooper, 1986; Atkinson et al., 1989; Bools et al., 1990; Dube and Orpinas, 2009). Many researchers historically have gravitated toward conclusions of dimensionality to describe this population (e.g., Rubenstein and Hastings, 1980; Kolvin et al., 1984; Hersov, 1985).

More specifically, meta-analytic and large-scale studies reveal broad, extensive overlap of internalizing and externalizing symptoms, absence types, and interventions for school refusal and truancy (Egger et al., 2003; Finning et al., 2018, 2019; Maynard et al., 2012, 2018). Neither pathognomonic nor reliable assident factors associated with the constructs have been identified, which often leads to interchangeable use of the terms in research and clinical practice (Brandibas et al., 2004). Contemporary notions of school refusal and truancy address these concerns to a degree (Heyne et al., 2019), though commonalities remain, such as tantrums, physical symptoms, reluctance or refusal to attend school, depression, sleep problems, variability in school attendance, and parental desire to have a child back in school.

Third, in related fashion, a school-refusal truancy distinction tends to erode in value at the point of clinical presentation. In the modern technological age, many parents are informed immediately of a child’s school absence, diminishing the value of distinguishing absenteeism based simply on parental knowledge or even consent (Smythe-Leistico and Page, 2018). Some parents are also skilled at securing medical notes or other methods to induce schools to record absences as excused in nature (Kearney, 2019). In addition, many children initially miss school due to anxiety but are later drawn to the amenities of staying home, and many adolescents who have been out of school for some time experience spikes in anxiety upon initial reintegration to school. Indeed, many youth described with school refusal or truancy traverse frequently between these groups (Birioukov, 2016). Clinicians are thus often faced with the challenge of choosing the best intervention for a child’s school attendance problems that appear to be of various types (Maynard et al., 2013; Kearney and Albano, 2018).

Finally, the concept of truancy carries with it many negative connotations that are not necessarily ascribed to concepts such as school refusal. Truancy is often used as a legal or institutional term, whereas school refusal is not, which may create stigmatization problems (Campbell and Wright, 2005; Strand, 2014). Indeed, anxiety-related school refusal may be viewed more sympathetically by school staff than truancy (Finning et al., 2019) and the label of truancy is often associated with willful, deliberate, deviant behavior (Lyon and Cotler, 2007; Birioukov, 2016). Educational and mental health agencies often emphasize the concept of truancy (in some form) in their definitions and discussions of problematic school absenteeism, but rarely that of school refusal or related terms (Gleich-Bope, 2014).

In related fashion, the overall concept of truancy has been criticized as representing more of a punitive paradigm that disproportionately affects vulnerable and at-risk youth and that contributes to the school-to-prison pipeline (Mallett, 2016; Nauer, 2016). The concept of truancy also tends to be associated with lower socioeconomic youth who experience barriers to attending school such as domestic and neighborhood violence, unstable housing conditions, lack of school supplies, housing and transportation problems, and safety concerns coming to school (Flaherty et al., 2012; Gottfried, 2017). Others view truancy less as an aberrant behavior than as a form of systemic discrimination that reflects the uneven distribution of social goods and opportunities within a larger society (Yang and Ham, 2017); others see truancy as deliberate student resistance against an unfair academic system (McIntyre-Bhatty, 2008).

### Excused-Unexcused Absences

Many school districts and some researchers also utilize an excused-unexcused absences dichotomy to categorize school attendance problems (Hough, 2019). Key advantages of this approach include its administrative practicality and simplicity, linkage to district and state policies regarding excessive absenteeism, historical connection (unexcused absences) to truancy, and utility in examining ratios of excused to unexcused absences (Gottfried, 2009). In addition, some have found that students absent without permission display approximately twice the odds of engaging in risky behaviors (e.g., unintentional injuries and violence, substance use, sexual behaviors) than students absent with permission (Eaton et al., 2008). Others have found that anxiety and depression symptoms are good predictors of unexcused absences in sexual minority youth (Burton et al., 2014).

An excused-unexcused absence dichotomy has several disadvantages, however. Numerous studies have illustrated ancillary problems associated with school absenteeism whether excused or unexcused, combine these absences when evaluating outcomes, or have found few differences based on this absence typology (Baker and Jansen, 2000; Redmond and Hosp, 2008; Spencer, 2009; Wood et al., 2012; Morrissey et al., 2014). For example, Gottfried (2009) found that excused and unexcused absences were both significantly related to various demographic, academic, and behavioral variables. Dube and Orpinas (2009) similarly found no difference between excused and unexcused absences across various profiles of youth with school attendance problems. The fidelity of data collected by school districts in this regard remains problematic as well, particularly because the arbiter of whether an absence is excused or unexcused is typically a family member and sometimes not a parent (Birioukov, 2016; Conry and Richards, 2018). In addition, excused absences may include legitimate reasons such as illness but also institutional or questionable reasons such as court dates, school suspensions, family vacations, or minor health conditions accommodated by physician notes (Reid, 2007; Outhouse, 2012).

In addition, reliance on an excused-unexcused absence dichotomy, particularly within school districts, often delays intervention until some legal tripwire is triggered (e.g., 10 unexcused absences in a semester). Some have criticized this approach as a “wait to fail” process that can enhance risk for school dropout (Cramer et al., 2014; Kearney and Graczyk, 2014). Indeed, the importance of early intervention for school attendance problems is quite clear in the literature (McCluskey et al., 2004; Sutphen et al., 2010). From a clinical perspective, evaluating total amount of time missed from school for any reason for a particular case may be advisable (Kearney and Albano, 2018).

### School Withdrawal and School Exclusion

As mentioned earlier, other categorical distinctions for school absenteeism have focused on parent-initiated (school withdrawal) and school-initiated (school exclusion) reasons. Potential explanations for parent-initiated school withdrawal were noted earlier. School exclusion can refer to disciplinary practices administered for absenteeism and other behavioral infractions, which usually means a child is not allowed to attend classes for a set period of time (Parker et al., 2015). Suspension can be in-school, meaning a child is physically in the school building but not in class, or out-of-school, meaning a child is not allowed on the school campus until certain requirements (e.g., parent conference, time away) are met. In related fashion, expulsion refers to permanent, administrative separation from a particular school, which sometimes applies to very severe infractions and possibly absenteeism and sometimes in response to zero tolerance policies (Allman and Slate, 2011). Other exclusionary practices such as detention may be utilized as well. In addition, as noted earlier, others have focused on school exclusion as school-initiated absence that is unlawful or that represents lack of appropriate accommodations (Reid, 2010).

A key advantage of identifying school withdrawal and school exclusion in cases of absenteeism involves rapid identification of non-child-based reasons for nonattendance and thus alternative assignment of treatment resources (e.g., toward parents or working with school officials) (e.g., Daniels and Cole, 2010). However, school district policies that emphasize suspension and expulsion to address school attendance problems lead paradoxically to more dropout, delinquency, lag in academic achievement, and student involvement with the juvenile justice system (Suh et al., 2007; Stone and Stone, 2011; Monahan et al., 2014). In addition, school exclusion does not appear to differ among various clusters of youth with school absenteeism (Gallé-Tessonneau et al., 2019). Unlawful school exclusion is also vaguely defined, difficult to track, and easily reframed as lawful school exclusion (McCluskey et al., 2016).

School exclusion policies also tend to be disproportionately assigned to low-income and diverse students (Shabazian, 2015). As such, exclusionary disciplinary policies have come under harsh criticism and are increasingly being reviewed and de-emphasized in many districts (Perry and Morris, 2014; Curran, 2016). Alternative responses that include greater proximity to school could involve sanctions such as in-school suspension and school-based community service as well as restorative practices such as mentoring and remediation of academic difficulties (Haight et al., 2014; McNeill et al., 2016; Gregory et al., 2018).

### Acute-Chronic

Another common historical dichotomy has been to distinguish acute from chronic school absenteeism. Though variously defined, acute cases of absenteeism often refer to those lasting less than one calendar year, whereas chronic cases of absenteeism often refer to those lasting more than one calendar year, or at least across two or more academic years (Baker and Wills, 1978; Berg et al., 1985). Some also distinguish between self-corrective problems lasting less than 2 weeks and acute problems lasting 2–52 weeks (Kearney and Silverman, 1996; Mauro and Machell, 2019). An acute-chronic distinction has been linked as well to more immediate onset involving emotional distress, akin to school refusal, and more insidious onset involving conduct problems, akin to truancy (Pellegrini, 2007). As such, an acute-chronic distinction is sometimes associated with other historical dichotomies such as Type 1-Type 2, common-induced, and neurotic-characterological (Kearney, 2001).

A key advantage of an acute-chronic distinction is a quick delineation of length of an absenteeism problem, which can be generally associated with breadth of intervention needed to resolve the problem. In general, more lengthy cases of absenteeism require more complex intervention and with multiple parties than less lengthy cases (Thambirajah et al., 2008). Prognostic outcomes for youth with more lengthy absenteeism tend to be poorer than those with less lengthy absenteeism (Kearney et al., 2010). An understanding of a child’s developmental history regarding his or her school attendance problems has substantial clinical value as well (Veenstra et al., 2010). Disadvantages to an acute-chronic distinction include variable timelines posed by researchers and the need for more empirical data to support a particular timeline distinction (Kearney, 2003; Balfanz and Byrnes, 2012).

### Diagnostic Categories

Other categorical distinctions with respect to school absenteeism have involved attempts at diagnostic groupings. Such groupings often involve anxiety, mood, and disruptive behavior disorders, including some combination of these (Bernstein and Garfinkel, 1986; Last and Strauss, 1990; McShane et al., 2001; Kearney and Albano, 2004). Anxiety- and mood-based categories are sometimes clustered in some youth with school attendance problems, as are oppositional defiant and conduct problems (King et al., 2001). As such, these distinctions are sometimes applied or related to school refusal-truancy or acute-chronic distinctions (Ek and Eriksson, 2013). Prognosis may relate to a degree to specific diagnostic type in this population as well (Layne et al., 2003; McShane et al., 2004).

Diagnostic groupings are appealing to many researchers and clinicians, but considerable diagnostic heterogeneity is a hallmark of youth with school attendance problems (Kearney, 2007; Nayak et al., 2018). In addition, several studies indicate that many youth with school attendance problems have no psychiatric diagnosis at all (Egger et al., 2003; Kearney and Albano, 2004). School attendance problems are not formally listed as psychiatric disorders in most nomenclatures, though aspects of these problems are represented in separation anxiety disorder and conduct disorder (American Psychiatric Association, 2013). As such, diagnostic profiles in this population have not been linked extensively to intervention recommendations.

### Summary

Categorical and dichotomous approaches to school attendance problems have a rich scholarly history and have contributed substantially to the conceptualization of this population. In addition, such approaches are well inculcated into many legal statutes, school-based policies, and research frameworks regarding school absenteeism. Key challenges for categorical and dichotomous approaches to school attendance problems include the need to better account for the considerable heterogeneity of this population and to link specific intervention strategies to specific constructs. In addition, these traditional characterizations are becoming challenged in an era of virtual learning, distance-based classrooms, hybrid education, blended education (e.g., high school with community college or vocational training), and other forms of alternative approaches toward graduation or career/adult readiness (see also Part 2 of this review). Categorical and dichotomous approaches to school attendance problems also do not generally focus on promoting school attendance, instead adopting more of a tertiary approach.

## Dimensional Approaches

As mentioned earlier, researchers and others have also examined dimensional approaches to SA/A to try to better account for the fluidity, scalability, and complexity of these constructs. These dimensional approaches include a focus on conceptualizing various aspects of SA/A along continua or spectra to more fully capture the heterogeneity, variability, diversity, and mutability of this population. General dimensions to be discussed over the next sections include definition, tiers of prevention/intervention, risk and contextual factors, absenteeism severity, developmental and school levels, and functional profiles.

### School Attendance and Its Problems on a Definitional Continuum

**Figure 1:**
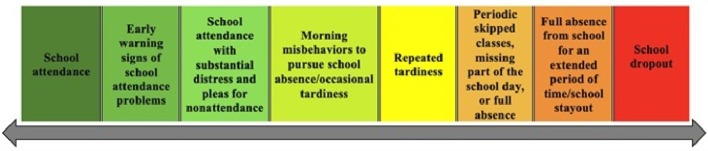
Spectrum of school attendance and its problems.

One of the most fundamental dimensional approaches to SA/A involves definition itself. This approach involves viewing school attendance and its various associated problems along a spectrum of panels ranging from full presence to complete absence (Figure 1). School attendance, with or without challenges or problems, generally represents the left side of the spectrum and can include attendance with little to no difficulty, early warning signs that may signal later absenteeism, school attendance under considerable distress, and morning misbehaviors designed to induce parental acquiescence or other responses that may eventually lead to absence from school (Kearney, 2019). Common early warning signs that may signal later absenteeism include frequent requests to leave the classroom or to contact parents, difficulties attending specialized sections of a school building (e.g., gymnasium, cafeteria), difficulties transitioning from class to class, persistent distress, and sudden changes in grades, completed work, or behavior, among others (Kearney and Graczyk, 2014).

The middle of the spectrum generally represents school attendance mixed with school absenteeism in some form, such as arriving late to school, missing some classes or times of day but not others, and periodic absences during a particular week, including early departures from school (Boylan and Renzulli, 2017). The right side of the spectrum represents complete school absenteeism, typically for an extended period of time in the form of school stayout (including school disengagement) or permanently in the form of school dropout (Iachini et al., 2016). The latter features of the spectrum account as well for the observation from many researchers that leaving school permanently is more of a process than an event (e.g., Ananga, 2011; Wang and Fredricks, 2014; Dupéré et al., 2015).

A key advantage of a dimensional approach to defining SA/A is that it includes the construct of school attendance and captures the full range of possible school attendance problems along a spectrum (Tobias, 2019). The spectrum allows for peri-attendance phenomena that are often fluid and change for a particular child over a certain time period (Chu et al., 2019; Kearney, 2019; Knollmann et al., 2019). For example, Pflug and Schneider (2016) found, among students with absenteeism in the past 7 days, that 35.0% missed a single class or part of a school day, 31.3% missed an entire day, and 33.7% missed 2+ days. In addition, the spectrum can account for the developmental history often surrounding SA/A in particular student, which can deteriorate over time in stages from full attendance to full absence (Henry et al., 2012). The spectrum is also largely atheoretical and may apply to various pathways to school dropout across countries (Lamb et al., 2010).

Such a dimension or spectrum allows for nimble, rapid, and real-time assessment of type of school attendance problem, which must be a priority for implementation models (see Part 2 of this review; Green et al., 2015). The dimension can also apply to variability in absenteeism that can exist between children in a given classroom, between classrooms in the same school, and between schools (Gee, 2019). The dimension also avoids pitfalls often associated with excused and unexcused absences by focusing more on type of school attendance problems and less on the need to establish the validity of an absence (Kearney and Albano, 2018). The dimension can apply as well to various tiers of SA/A (see “Multi-tiered System of Supports”).

Key drawbacks of the definitional spectrum include its lack of current utility in school districts and research studies, inability to provide information about the etiology or function of a school attendance problem, and lack of association with prevention or intervention protocols for this population (Schildkamp et al., 2016; Balfanz and Byrnes, 2018). Specific, operational definitions for each panel of the spectrum remain needed as well (Kearney, 2016). Others contend that collecting even very basic absenteeism data is challenging enough for many schools, and that basic data may be sufficient for at least determining which students are missing a substantial amount of school (Birioukov, 2016). Still, researchers commonly examine school attendance problems other than full absenteeism, clinicians and others must initially grapple with the exterior complexity of this population, and the spectrum can be a useful heuristic for understanding the full scope of school attendance and its problems across jurisdictions (Keppens and Spruyt, 2017; Kearney, 2019; Wegmann and Smith, 2019).

### Multi-tiered System of Supports

As noted earlier, the sheer number of disciplines associated with the study of SA/A has led to a plethora of intervention approaches to address this complicated population. Such approaches range from (1) systemic prevention strategies developed by educators and criminal justice experts to promote school attendance and curb dropout, (2) clinical approaches developed by health professionals to address mental health and other challenges during emerging school absenteeism, (including aspects described in the previous section), and (3) intensive strategies developed by professionals in multiple disciplines to address chronic and severe absenteeism and potential dropout often mixed with substantial, broad contextual factors related to extreme psychopathology, family crises, and school and community variables (Wilson et al., 2011; Freeman and Simonsen, 2015). An advantage of these varied set of approaches is as much a focus on promoting school attendance and preventing school attendance problems as on ameliorating existing cases of school absenteeism (Ekstrand, 2015).

Kearney and Graczyk (2014, see also Kearney, 2016) advocated the use of multi-tiered system of support principles to arrange extant strategies to boost school attendance and to address school absenteeism at different severity and risk/contextual factor levels. Multi-tiered system of support (MTSS) models have been utilized in education for many years and typically weave the academic focus of Response to Intervention (RtI) models and the behavioral and social focus of positive behavior intervention supports (PBIS) or program-wide positive behavior supports (PWPBS) into one cohesive model to best address all student needs (Sugai and Horner, 2009). An overarching principle of MTSS is to eschew a “wait to fail” mentality and to instead emphasize active monitoring and more immediate intervention (McIntosh and Goodman, 2016). MTSS models thus accentuate prevention, frequent progress monitoring, data-based decision-making and problem-solving, evidence-based interventions, individualized instruction and intervention, and implementation fidelity (Eagle et al., 2015). The comprehensive, empirical, sustainable, and efficient nature of MTSS is designed to optimize limited resources and is thus becoming widely adopted in school settings (McIntosh et al., 2010; August et al., 2018).

MTSS models commonly arrange prevention and intervention strategies for a particular problem (or non-problem) into three tiers: primary or universal (Tier 1), secondary or targeted (Tier 2), and tertiary or intensive (Tier 3) (Stephan et al., 2015; Stoiber and Gettinger, 2016). Tier 1 strategies involve delivering support to all students and are generally designed to promote a positive school culture and prosocial behavior and academic competence and to prevent difficulties in these areas. Tier 2 strategies involve delivering support to a percentage of students who do not respond in some way to Tier 1 strategies but who have less complex concerns. Tier 3 and more individualized strategies involve delivering support to a lesser percentage of students who do not respond in some way to Tier 2 strategies and who have more complex concerns (Rodriguez et al., 2016). The tiers represent a continuum of evidence-based practices implemented by various teams (Cook et al., 2015; Weist et al., 2018).

**Figure 2:**
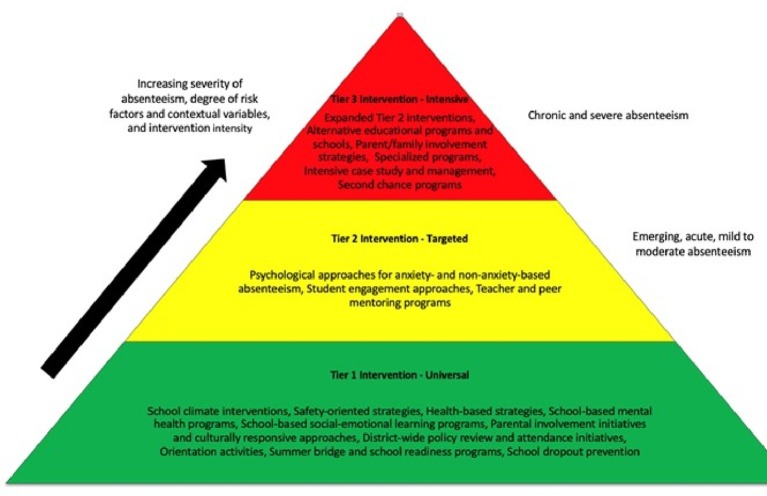
A multi-tiered system of supports model for SA/A.

Kearney and Graczyk (2014) initially focused on RtI descriptives for arranging strategies that promote school attendance and address school absenteeism, and Kearney (2016) later expanded this line of thinking to broader MTSS descriptives. The essential aspects of each are similar for this population: Tier 1 approaches focus on enhancing functioning and school-wide attendance and on preventing absenteeism for all students, Tier 2 approaches focus on addressing students with emerging, acute, or mild to moderate school absenteeism, and Tier 3 approaches focus on addressing students with chronic and severe school absenteeism (Kearney, 2016, 2019; Fornander and Kearney, submitted). Tiers 2 and 3 would thus include the definitional spectrum discussed in the previous section. Specific preventative-based and clinical and systemic interventions are matched to each tier to help school personnel and others conceptualize approaches to SA/A. Figure 2 illustrates a sample MTSS model for SA/A prevention/intervention.

An MTSS model for SA/A includes several dimensions designed to enhance inclusivity, flexibility, and adaptability to various disciplines, educational and health structures, and jurisdictions and possibly cultures. These dimensions include severity of absenteeism (e.g., percentage days missed in a given year, length of problem; see previous section), degree of risk or contextual factors present in a particular case (i.e., child, parent, family, peer, school, community), target of prevention/intervention (i.e., all students, some percentage of students, fewer percentage of students), and intensity and breadth level of interventions (e.g., less intense/broad for acute or mild to moderate absenteeism, more intense/broad for chronic and severe absenteeism). At the same time, however, an MTSS model for SA/A is designed to be fairly simple in scope to be more easily adapted to various individual cases and settings. The model is thus, essentially, a signpost or roadmap to chart available intervention strategies for SA/A.

A full description of preventative and intervention approaches to SA/A is beyond the scope of this article. In general, however, Tier 1 approaches for SA/A can include system-, district-, school-, or even community-wide or state/national approaches to promote school attendance and prevent school absenteeism, often in tandem (e.g., full service community schools; Coffey et al., 2018). These approaches are generally aimed at all students and may include methods to improve school climate and safety, to enhance mental and physical health and social-emotional functioning, to boost parent and family involvement, to reduce school violence and bullying, to review policies that may exacerbate attendance problems, and to implement orientation and readiness programs, among others (see comprehensive summaries by Sutphen et al., 2010; Maynard et al., 2013, 2018; Kearney, 2016). Similarly, school dropout prevention efforts typically focus on school-wide academic enhancement, mentoring and supportive relationships, psychosocial skill development, and effective classroom behavior management (Ecker-Lyster and Niileksela, 2016). Many of these Tier 1 approaches have been shown to improve school attendance rates, and reduce school dropout rates, either directly or indirectly (e.g., Havik et al., 2015; Freeman et al., 2016; Taylor et al., 2017).

Tier 2 approaches for SA/A can include child-, parent-, and family-based interventions for cases of emerging, acute, or mild to moderate school absenteeism severity. These approaches are generally aimed at the percentage of all students/families who display these problems and may include the many psychological and psychiatric interventions designed for this population as well as approaches to enhance individual student engagement and school connectedness (Estell and Perdue, 2013; Maynard et al., 2013, 2018; Kearney, 2019). Mentoring and monitoring approaches may be relevant in this regard as well (Guryan et al., 2017). Many of these Tier 2 approaches can be and have been adapted as well for more severe cases of school absenteeism (i.e., Tier 3) (Heyne et al., 2002), but many Tier 2 approaches tend to work better for cases of less severe absenteeism with fewer complicating factors (Kearney, 2016).

Tier 3 approaches for SA/A can include various system-wide school-community partnerships as well as individual approaches to address cases of chronic and severe absenteeism (Kim and Streeter, 2016). These partnerships and approaches are generally aimed at the smaller percentage of all students/families who display these problems and may include alternative educational placements and opportunities, individualized efforts to re-engage parents and family members in the educational/attendance process, and specialized programs for youth with extreme psychopathology (Flower et al., 2011; Hahn et al., 2015; Kearney, 2016). A key aspect of many Tier 3 approaches to SA/A for secondary students is to focus not so much on traditional in-seat class time and formal credit accrual as much as on flexible avenues that blur the end of high school and the beginning of adult or career readiness paths such as community college, vocational training, or technical certification (Dougherty and Lombardi, 2016). As such, many approaches for this population focus more on demonstration of competencies than on traditional metrics such as grades (Castellano et al., 2017).

An MTSS approach to SA/A remains in development and will likely need to evolve in conjunction with related progressions in the field. For example, some have advocated for moving beyond one-dimensional triangle representations of MTSS to more multifaceted pyramids, with each side of the pyramid addressing a different type of student (Dulaney et al., 2013) (see Part 2 of this review). Kearney (2016) also discussed the idea of a “Tier 4” for youth with extreme psychopathology and the need for inpatient/residential treatment mixed with education. How an MTSS approach for SA/A fits with related approaches focused on academic, behavioral, and social constructs also remains to be seen, especially given that absenteeism rates in some schools (and thus entry into Tiers 2 and 3) are overwhelming (Balfanz et al., 2014).

Still, schools that implement MTSS with higher fidelity have less school absenteeism than schools that implement with less fidelity (Freeman et al., 2016). School districts may also include attendance measures in MTSS models (Coffey et al., 2018). Others have also begun to utilize a general tiered framework to place their studies and interventions in this context (e.g., Skedgell and Kearney, 2018; Brouwer-Borghuis et al., 2019; Elliott and Place, 2019; Ingul et al., 2019). For example, Cook et al. (2017) evaluated a comprehensive program to reduce school attendance problems that included components of each tier of intervention. Tier 1 involved facilitating communication between teachers and parents *via* home visits and mobile telephone contact, Tier 2 involved attendance data monitoring and teacher intervention with students beginning to accrue excessive absences, and Tier 3 involved referrals to specialists for students with chronic absenteeism. A multidimensional MTSS framework will comprise a key piece for reconciling SA/A approaches in Part 2 of this review.

### Risk/Contextual Factors, Absenteeism Severity, and Developmental Level

As mentioned, key dimensions of an MTSS model of SA/A involve risk and contextual factors, which are generally expected to accrue by tier in conjunction with greater absenteeism severity. Researchers commonly group risk or contextual (and, conversely, protective) factors for SA/A into various categories that include child-, parent-, family-, peer-, school-, and community-based variables (Kearney, 2008; Zaff et al., 2017; Gubbels et al., 2019). Others have argued that broader societal or cultural variables also impact school attendance problems, including zero tolerance-based legal statutes, assimilation and language barriers, and immigration issues, among others (Casoli-Reardon et al., 2012). Categories of risk and contextual factors for SA/A are sometimes studied singularly (e.g., Hendron and Kearney, 2016), though many recent approaches have utilized more sophisticated multilevel modeling and related statistical procedures to examine these categories collectively (Dembo et al., 2016; Van Eck et al., 2017; Ramberg et al., 2019). An accumulation of risk/contextual factors appears to exacerbate risk of school attendance problems (Catalano et al., 2012; Ingul et al., 2019) and thus may be more evident in Tier 3 than Tier 2 cases (Vaughn et al., 2013).

Similarly, absenteeism severity is an important dimension of an MTSS model of SA/A and can be generally measured as percentage days missed from school in a given academic year (Fornander and Kearney, submitted). However, this dimension can also be more broadly conceptualized as developmental history of a child’s SA/A across multiple academic years (Veenstra et al., 2010). Risk and contextual factors as well as absenteeism severity can also change along a continuum of developmental and school levels (Skedgell and Kearney, 2018). Risk factors for school absenteeism can manifest quite differently across primary, early secondary, and later secondary grades (Suh and Suh, 2007). In addition, absenteeism severity rates in schools tend to spike in kindergarten and first grade, decline during elementary school years, spike again in middle school, and continue to increase through high school, peaking at 12th grade (Balfanz and Byrnes, 2012).

### Functional Profiles of School Attendance Problems

Many schools and school-based professionals that utilize tiered frameworks for academic, behavioral, and social issues also rely heavily on functional analysis and functional behavioral assessment practices to provide individualized student support (Simonsen and Sugai, 2013; McCurdy et al., 2016). At Tier 1, this may include a focus on school-wide antecedents or predictors of problem behavior, delineating appropriate and nuanced consequences for a behavior depending on its function and severity, and adjusting expectations across contexts and personnel (Crone et al., 2015). At Tier 2, this may include selecting and monitoring social and behavioral interventions for students on the basis of the function of their behavior (Reinke et al., 2013). At Tier 3, this may include a more detailed assessment of multiple functions and replacement behaviors as well as more complex environmental change (Scott and Cooper, 2013).

Kearney and colleagues (e.g., Kearney and Silverman, 1996; Kearney and Graczyk, 2014; Gonzalvez et al., 2019) developed various aspects of a functional model of school attendance problems designed to apply particularly to school refusal behavior (i.e., child-initiated school attendance problems). This model focuses on key variables or functions that serve to maintain or reinforce school attendance problems and was designed primarily as a clinical approach for Tier 2-type school attendance problems. The postulated primary functions in the model include refusal to attend school to (1) avoid school-based stimuli that provoke a general sense of negative affectivity (i.e., aspects of both anxiety and depression), (2) escape aversive social and/or evaluative situations at school, (3) seek attention from significant others such as parents, and/or (4) pursue tangible rewards outside of school such as time with friends.

The first two functions refer to school refusal behavior maintained by negative reinforcement, whereas the latter two functions refer to school refusal behavior maintained by positive reinforcement. A profile of the relative strength of each functional condition is generally recommended during case analysis (Kearney, 2019). A key advantage of the functional model is its clear linkage to specific prescriptive treatment packages that include child-, parent-, and family-based interventions as well as Tier 3 interventions as needed (Kearney and Albano, 2018). The treatment packages are also designed to be flexible enough to be adapted to a variety of cases and locations, and indeed have been across educational, mental health, and medical settings (e.g., Tolin et al., 2009; Rohrig and Puliafico, 2018; Hannan et al., 2019; Thastum et al., 2019).

Another key aspect of the functional model is its amenability to support the study of various dimensions or profiles of youth with school attendance problems. Researchers have demonstrated across numerous studies that functions of school refusal behavior relate to different patterns of depression, anticipatory and school-based performance anxiety, stress, positive/negative affect, sleep problems, and social functioning (e.g., Kearney, 2002; Richards and Hadwin, 2011; Hochadel et al., 2014; Fernández-Sogorb et al., 2018; Gonzálvez et al., 2018; Sanmartín et al., 2018; Gonzalvez et al., 2019). Others have related the functions to clusters of absentee youth (Gallé-Tessonneau et al., 2019) and family environment types (Kearney and Silverman, 1995). In addition, functions of school refusal behavior may be superior to forms of behavior in predicting absenteeism severity (Kearney, 2007).

A functional model of school refusal behavior does carry limitations, however. As noted, the model is meant to apply primarily to Tier 2 (and perhaps to early warning signs evident in Tier 1) school refusal behavior and thus less to more chronic and severe school absenteeism or to cases primarily initiated by other entities (Kearney, 2016). In addition, the model is not necessarily applicable to all countries and cultures, though many have found analogous features in their locales (e.g., Brandibas et al., 2004; Kim, 2010; Secer, 2014). In addition, some erroneously conflate specific assessment devices constructed to assist the functional model with the broader model itself, which is supposed to be based on a comprehensive analysis of maintaining variables (Kearney and Tillotson, 1998).

### Summary

Dimensionally oriented approaches to SA/A may help account for the considerable heterogeneity of this population by capturing a wide range of attendance/absenteeism expressions, prevention and intervention strategies, risk/contextual factors, absenteeism severity and developmental levels, and functional profiles of key maintaining factors. Dimensional approaches do consider school attendance as much as absenteeism and are helpful in informing treatment approaches for SA/A. As with categorical approaches, however, considerable barriers exist to implementing dimensional approaches in schools and other pertinent settings. In addition, dimensional approaches to SA/A will also have to adapt to rapid advancements in education and technology in future years.

## General Summary

The plethora of conceptual approaches to SA/A is certainly a phenomenon worth celebrating. Researchers, educators, clinicians, and stakeholders such as parents have contributed immensely to the study and understanding of this complex population. Such study has involved definitions, classification systems, assessment protocols, and intervention strategies designed, in the end, to help children and adolescents attend school and to achieve better outcomes in adulthood. We salute all of those who have dedicated their time and careers to improving the lives of these students.

Part 1 of this two-part review concentrated on a broad classification and description of contemporary approaches to SA/A along categorical and dimensional orientations. Each orientation carries distinct advantages and disadvantages, a not uncommon circumstance across various problems and disorders that affect youth. Though meant to be comprehensive, this review focused on the primary methods of differentiating school attendance problems. Many nuanced distinctions based on multilevel and other statistical modeling should be noted, and many special circumstances such as intense school violence or extreme poverty likely override the distinctions mentioned here. In addition, prevention and intervention were not a primary focus of this part of the review, but are explored in greater depth in the second part of this review.

As suggested by several scholars, adopting both categorical and dimensional approaches to the study of complex and heterogeneous phenomena may be advisable. Such a juxtaposition has the potential advantage of identifying general categorical rules and cut-points for distinguishing broad groups of behavior as well as specific dimensions that are useful for providing data to adjust these cut-points along various spectra. Part 2 of this two-part review thus focuses on a possible pathway toward reconciling contemporary categorical and dimensional approaches to SA/A in this manner. This pathway also represents a heuristic framework as the field of SA/A grapples with challenges to dissemination and implementation as well as future changes in education and technology.

## Author Contributions

CK, CG, PG, and MF contributed to the writing and editing of the manuscript.

### Conflict of Interest

The authors declare that the research was conducted in the absence of any commercial or financial relationships that could be construed as a potential conflict of interest.
