# Erratum: Reconciling Contemporary Approaches to School Attendance and School Absenteeism: Toward Promotion and Nimble Response, Global Policy Review and Implementation, and Future Adaptability (Part 1)

**DOI:** 10.3389/fpsyg.2019.02766

**Published:** 2019-11-27

**Authors:** Frontiers Production Office

**Affiliations:** Frontiers Production Office, Frontiers Media SA, Lausanne, Switzerland

**Keywords:** school attendance, school absenteeism, truancy, school refusal, school withdrawal, school exclusion, multi-tiered system of supports, response to intervention

Due to an error in the typesetting process, the short running title was incorrectly provided as “Dairy Manure and Rasperry Safety.” The correct short running title is “School Attendance and School Absenteeism.”

The publisher apologizes for this mistake. The original article has been updated.

